# Clinical outcomes of pars plana vitrectomy with endoresection in uveal melanoma primarily treated with plaque radiotherapy or proton beam therapy

**DOI:** 10.1177/11206721251400512

**Published:** 2025-12-17

**Authors:** Leonoor S Koetsier, Marina Marinkovic, Jaco C Bleeker, Coen RN Rasch, Khanh THK Vu, Gregorius PM Luyten, Thomas J van Rijssen

**Affiliations:** 1 Department of Ophthalmology, 4501 Leiden University Medical Center, Leiden, the Netherlands; 2 Department of Radiotherapy, 4501 Leiden University Medical Center, Leiden, the Netherlands

**Keywords:** Uveal melanoma, endoresection, ruthenium brachytherapy, proton beam therapy

## Abstract

**Background:**

To describe the characteristics and outcomes of endoresection in patients with uveal melanoma previously treated with ruthenium brachytherapy or proton beam therapy.

**Methods:**

In this retrospective study, data on the indication to perform endoresection, best-corrected visual acuity, tumor characteristics, surgery details, and characteristics of ruthenium brachytherapy or proton beam therapy were obtained.

**Results:**

A total of 27 patients (12 men and 15 women) were included, with a median age of 63.5 (range 50.5) years. The indication for endoresection was exudative retinal detachment in 20 patients, glaucoma induced by tumor bleeding in 6 patients, and persistent inflammation in 1 patient. Ruthenium brachytherapy was performed in 6 patients, while 21 patients received proton beam therapy. In the ruthenium group, mean visual acuity before endoresection in Snellen was 0.099 ± 0.197, which increased to 0.285 ± 0.302 after endoresection (p = 0.043). In the proton beam group, mean visual acuity before endoresection was 0.030 ± 0.044, which decreased to 0.020 ± 0.036 after endoresection (p = 0.546). Mean change in visual acuity before and after endoresection was +0.186 ± 0.249 in the ruthenium group and −0.009 ± 0.053 in the proton beam group. Enucleation had to be performed in 1 eye in the ruthenium group and 8 eyes in the proton beam group. No surgery-related vascular events were observed.

**Conclusions:**

Patients previously treated with ruthenium brachytherapy have favorable post-endoresection outcomes, opposed to those treated with proton beam therapy. Patient-specific counselling is important before endoresection, as there is a wide variety in expected outcomes.

## Introduction

Uveal melanoma is the most common primary intraocular malignancy in adults. Treatment of uveal melanoma includes plaque brachytherapy, (proton-beam) radiotherapy, (endo)resection, or enucleation. Plaque brachytherapy is often used in small to medium -sized uveal melanomas, while proton beam radiotherapy is more common in larger tumors or those tumors that are inaccessible to plaque brachytherapy (for example close to the optic nerve).^1^ The affected eye is enucleated when the tumor size is substantial and/or beyond indication of local treatment, such as extensive involvement of the optic disc, when the risk of radiotherapy-related side effects is high, and patient-specific preferences. Primary endoresection has also been performed in cases which are susceptible to radiation- induced optic neuropathy. However, this technique is controversial because of the risk of local recurrences and consequent metastases induced by tumor progression.^2–4^ To reduce this risk, neoadjuvant irradiation has been proposed by several investigators.^5–7^

While tumor control is the primary goal in the improvement of eye-sparing treatments and survival, outcomes such as visual acuity are becoming more important. A visual acuity of 20/40 can be retained in 39–55% of patients at 4–9 years after treatment with ruthenium brachytherapy.^8,9^ After treatment with proton beam radiotherapy, a visual acuity of 20/40 or more has been reported in 23% of patients after a median of 32 months follow-up.^10^ Availability of both ruthenium brachytherapy and proton beam therapy at the treatment center may also affect visual outcomes.^1^ Pre-treatment visual acuity, tumor thickness, and involvement of the macula have been identified as risk factors for vision loss.^11^ However, even when the tumor is located near or in the fovea, patients treated with proton beam therapy can retain a usable visual acuity.^12^

Post-treatment visual function is affected by several characteristics, such as the presence of an exudative retinal detachment, vitreous hemorrhage, or other post-radiation effects.^13^ These characteristics may be present before treatment or develop over time after treatment. Pars plana vitrectomy with endoresection of the tumor may be performed in order to improve visual acuity and/or prevent secondary enucleation. This study reports the clinical outcomes of pars plana vitrectomy with endoresection in patients with a retinal detachment or vitreous hemorrhage after ruthenium brachytherapy or proton beam therapy. Endoresection was performed as a secondary procedure to treat cases with post-radiation eye disease.

## Methods

This retrospective study included patients with uveal melanoma previously treated with ruthenium brachytherapy or proton beam therapy, who underwent pars plana vitrectomy with endoresection of the tumor. This study adhered to the tenets of the Declaration of Helsinki, and approval was acquired from the institutional review board. The medical ethics committee waived the need for their approval and informed consent due to the retrospective nature of this study.

Medical records of all consecutive patients undergoing pars plana vitrectomy with endoresection after previous treatment of a uveal melanoma with ruthenium brachytherapy or proton beam therapy between April 2020 and August 2023 were examined. Patients had to be 18 years or older to be included. Data on age, sex, date of ruthenium brachytherapy or proton beam therapy, date of endoresection, visual acuity before and after treatment, and possible enucleation were obtained. Ultrasound (b-scan) data was reviewed to determine the largest tumor diameter and thickness.

The recommendation whether to treat the tumor with ruthenium brachytherapy or proton beam therapy was discussed in a multidisciplinary setting, including ocular oncologists and radiation oncologists. The criteria included the tumor diameter, tumor prominence, and location of the tumor. Individual patient characteristics and preferences were also taken into account.

### Ruthenium brachytherapy

Tumor diameter and prominence including sclera were measured using b-scan ultrasonography (Absolu, Quantel Medical, Cournon-d'Auvergne, France) and used to determine the treatment dose required for ruthenium brachytherapy (Ru-106). Radiation dose was specified at the largest depth to the tumor apex, taking the plaque-specific characteristics and duration of the application into account.

Ru-106 applicators (Bebig, Germany) of 17.9 mm (CCD) or 20.2 mm (CCB) were used in this study.^14^

The type of the applicator was chosen in a multidisciplinary setting by the ocular oncologists and radiation oncologists. During surgery, a dummy was sutured to the sclera to ensure the correct localization of the radioactive applicator over the tumor with a minimal safety margin of 2 mm in all directions. Finally, the conjunctiva was sutured over the plaque. The plaque was removed at the calculated time with a 5% margin.

### Proton beam therapy

Prior to proton beam therapy, four tantalum clips were sutured to the sclera. The distances between the tumor and between the clips were measured intraoperatively using funduscopy and diaphanoscopy. A model was created based on Biometry, Ultrasound imaging, per-operative measurements, MRI imaging, clip-clip, and clip-tumor distances, in order to determine the radiation route. A dose of 4 × 15 Gy was used.

### Endoresection

Endoresection was proposed to patients with exudative retinal detachment, persistence of tumor hemorrhage, neovascular glaucoma with tumor hemorrhage, or persistent inflammation. Surgery was planned 6 weeks after diagnosis. Three-port pars plana vitrectomy and endoresection (23 gauge, Constellation system, Alcon, United States of America) was performed by an senior retinal surgeon (LK) under hypotensive general anesthesia. The technique largely resembles the procedure described previously by Damato et al.^2^ A twin light (DORC, The Netherlands) was used for visualization and tumor debulking was achieved with a vitrectome, and in some cases with help of scissors and forceps. Obtained material was sent to the department of pathology. Hemostasis was achieved by raising the intraocular pressure (up to 80 mmHg) and endodiathermy if necessary. Reattachment of the retina was attained with C10H18 (decalin), with precautions to prevent embolisms. These precautions included optimal hemostasis and reassessment of a proper positioning of the infusion canula before fluid-air exchange, and minimizing air exposure (by doing partial fluid-air exchange up to the endoresected area, or direct decalin-oil exchange) and intraocular pressure during the procedure.^15–17^ Consequently, endolaser was used to achieve retinal adhesion around the tumor site. Triamcinolone (Kenacort) and methylcellulose (Celoftal) were administered at the surgeon's discretion. Silicone oil (1000 or 5000 centistokes (cst)) or gas (SF6 or C3F8) was used for tamponade. Sclerotomy ports were sutured with 8-0 polyglactine 910 (Vicryl, Ethicon). The obtained specimens were sent to the pathology department for genetic status and tumor activity assessment. Patients were discharged at the same day and prescribed tobramycin/dexamethasone (Tobradex) eyedrops 4 times a day (decreasing one drop every week) and broomfenac (Yellox) eyedrops twice daily up to a month after surgery. Patient were examined the next day, one week, and six weeks after surgery, and additionally if deemed relevant.

### Statistical analyses

The statistical analyses were performed with SPSS Statistics (IBM Corp. version 29.0. Armonk, New York, United States of America). Kolmogorov-Smirnov and Shapiro-Wilk tests were used to test normality. Chi-square test, T-test, Mann-Whitney U test, Wilcoxon signed rank test, and Fishers exact test were used for the analyses depending on whether the data was normally distributed.

## Results

**Figure 1.**
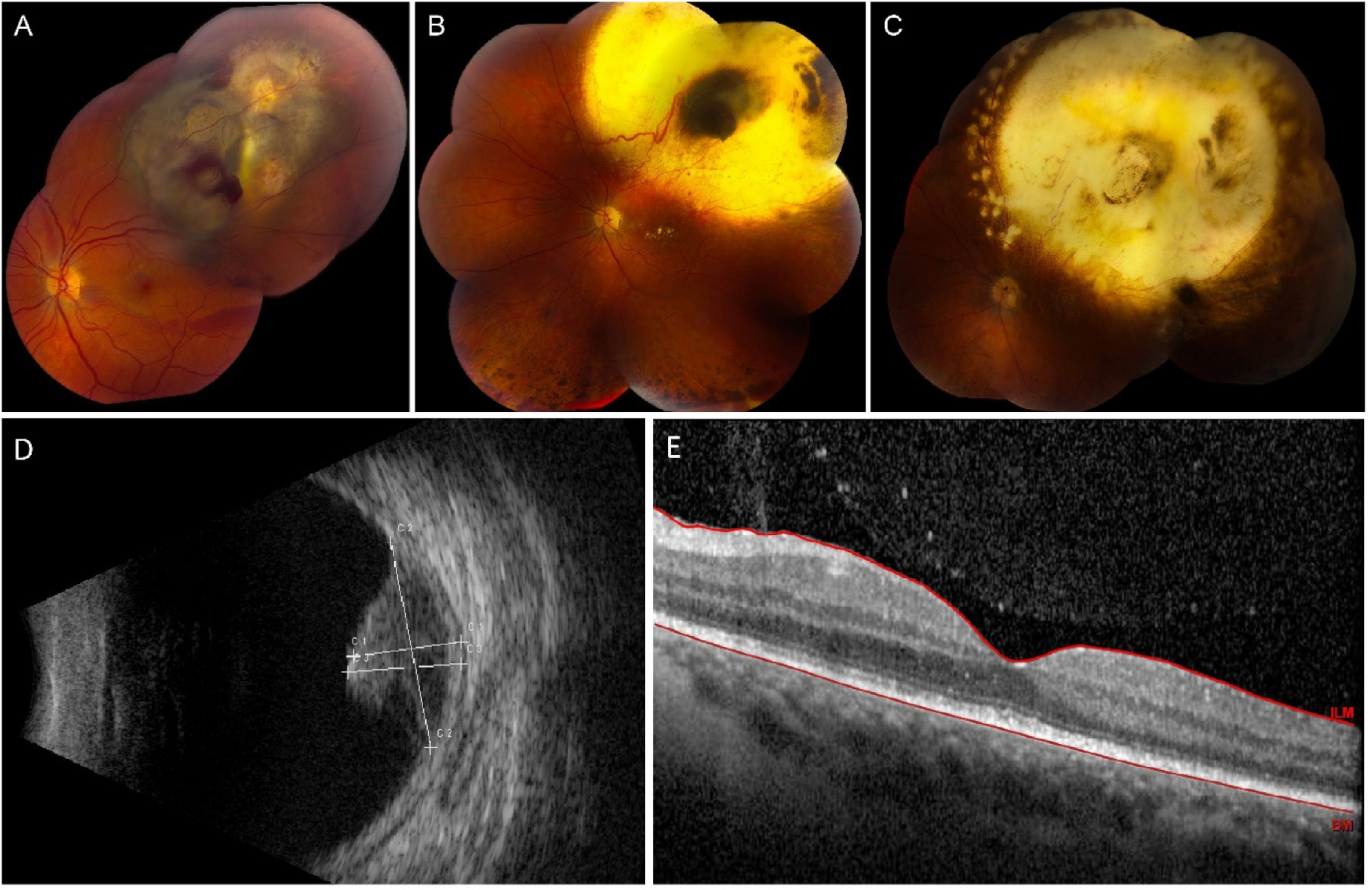
Fundus photography of a 33 year old man diagnosed with a choroidal melanoma in the left eye (A, B, C). This patient received transpupillary thermotherapy after ruthenium brachytherapy because of recurrent vitreous hemorrhages. Vitrectomy with endoresection was performed because of additional recurrences. A choroidal melanoma with hemorrhage, atrophy, and pigment alterations are present before ruthenium brachytherapy (A), without macular involvement on optical coherence tomography (E). On ultrasound bio microscopy, a tumor thickness of 7.09 millimeters was measured (D). After ruthenium brachytherapy, a feeder vessel and pigmented center surrounded by retinal atrophy can be observed, along with exsudates in within the macular area (B). Ultrasound bio microscopy showed a tumor thickness of 3.64 millimeters. After endoresection, the feeder vessel and pigmented center are less apparent (C). Laser coagulates can be appreciated at the nasal, superior, and temporal side of the area of retinal atrophy (C). There was no thickness on ultrasound bio microscopy after endoresection.

A total of 27 eyes of 27 patients (12 men and 15 women) with uveal melanoma previously treated with ruthenium or proton therapy who underwent pars plana vitrectomy with endoresection could be included. All patients were of Caucasian descent. Multimodal imaging of a patient treated with ruthenium and consequently endoresection is depicted in Figure 1. The descriptives of both the ruthenium and proton beam group are summarized in Table 1.

**Table 1.** Characteristics of uveal melanoma patients treated with ruthenium brachytherapy or proton beam therapy and subsequent endoresection.

	Ruthenium brachytherapy (n = 6)	Proton beam therapy (n = 21)	P-value
Median age in years (interquartile range)	67.7 (16.5)	63.1 (15.4)	0.600
Mean largest tumor diameter in mm	12.5 ± 1.3	15.8 ± 3.0	0.016
Mean tumor thickness before radiation treatment in mm	5.7 ± 1.9	9.3 ± 2.0	<0.001
Mean tumor thickness before endoresection in mm	4.4 ± 1.6	9.7 ± 3.0	0.001
Mean time between radiotherapy and endoresection in months	26.0 ± 29.4	9.4 ± 11.6	0.042
Mean time from radiotherapy to last available visit in months	37.1 ± 33.0	19.7 ± 15.1	0.072
Mean visual acuity before endoresection in Snellen	0.099 ± 0.197	0.030 ± 0.044	0.597
Mean visual acuity after endoresection in Snellen	0.285 ± 0.302	0.020 ± 0.036	0.012
Mean change in visual acuity before and after endoresection in Snellen	+0.186 ± 0.249	−0.009 ± 0.053	0.003
mm, millimeter; ± standard deviation			

The median age was 63.5 (interquartile range 14.6) years at the date of radiation therapy. The indication for endoresection was exudative retinal detachment in 20 patients (of whom 3 in the ruthenium group), persistence of tumor hemorrhage in 5 patients (of whom 1 in the ruthenium group), neovascular glaucoma with tumor hemorrhage in 1 patient in the ruthenium group, and persistent inflammation in 1 patient in the ruthenium group. Before radiation treatment, the macula was involved (with tumor, retinal detachment or hemorrhage) in 13 out of 27 patients, while this was 24 out of 27 patients at the visit before endoresection. Ruthenium brachytherapy was performed in 6 patients, while 21 patients received proton beam therapy. Before treatment, the mean largest tumor diameter in millimeter was 12.5 ± 1.3 in the ruthenium group and 15.8 ± 3.0 in the proton beam group (p = 0.016). Mean tumor thickness before radiation treatment was 5.7 ± 1.9 millimeter in the ruthenium group and 9.3 ± 2.0 millimeter in the proton beam group (p < 0.001). Mean tumor thickness before endoresection was 4.4 ± 1.6 millimeter in the ruthenium group and 9.7 ± 3.0 millimeter in the proton beam group (p = 0.001). The differences in size and tumor thickness can be attributed to the treatment selection criteria.

Mean time between ruthenium therapy and endoresection was 26.0 ± 29.4 months, while mean time between proton beam therapy and endoresection was 9.4 ± 11.6 months (p = 0.042). Mean time from ruthenium treatment to last available visit was 37.1 ± 33.0 months and from proton beam therapy to last visit was 19.7 ± 15.1 months (p = 0.072). The time from date of endoresection to last available visit ranged from 6 to 914 days.

After endoresection, silicone oil 1000 cst was used in 14 eyes, silicone oil 5000 cst in 5 eyes, SF6 gas in 2 eyes, and C3F8 gas in 3 eyes. The surgery did not have the anticipated treatment outcome (acceptable intraocular pressure and reattachment of the retina) in 3 eyes, including 2 eyes due to persistent hemorrhage and 1 eye with retinal adhesions. None of the patients had embolism-related events during or directly after endoresection. At the last available visit after endoresection, visual acuity had improved in 14 eyes, decreased in 11 eyes, and remained stable in 2 eyes. Enucleation was performed in 1 eye in the ruthenium group and 8 eyes in the proton beam group during available follow-up, which was due to complete retinal detachment (2 eyes, 1 in the ruthenium group and 1 eye in the proton beam group), uncontrollable intraocular hemorrhage (4 eyes), neovascular glaucoma (2 eyes), or extensive retinal adhesion (1 eye). Time between endoresection and enucleation varied from 0 to 10 months (one endoresection was converted to enucleation because of persistent intraocular bleeding). During available follow-up, liver metastases were observed in 1 patient (17%) in the ruthenium group, and in 5 patients (24%) in the proton beam group (p = 0.596).

In all patients the diagnosis of uveal melanoma was confirmed by pathological examination of material obtained during endoresection or enucleation. Loss of BAP1 was observed in 13 eyes, no loss of BAP1 was observed in 10 eyes, and in 4 eyes BAP1 status was unknown.

**Figure 2.**
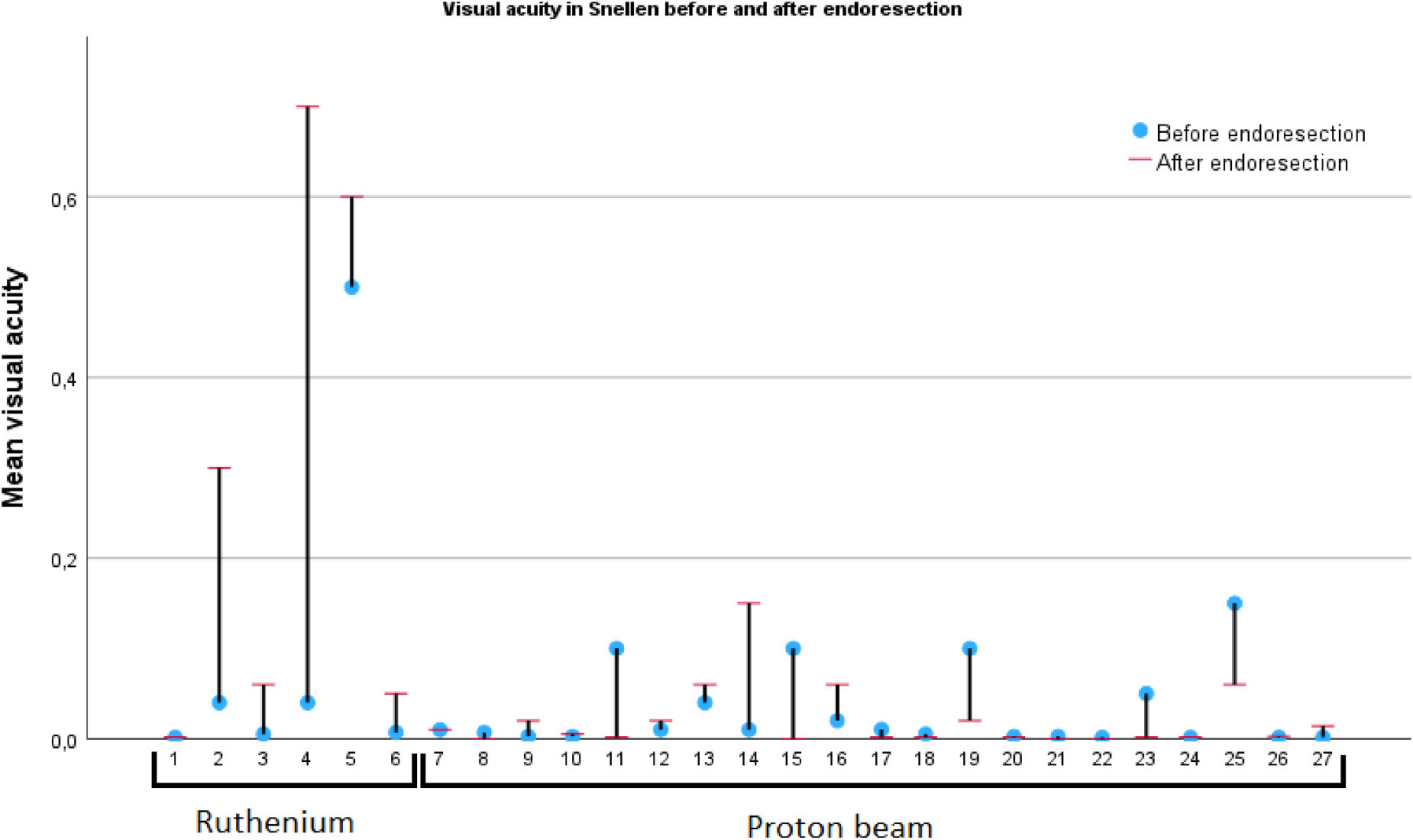
Best-corrected visual acuity before and after endoresection in uveal melanoma patients previously treated with ruthenium brachytherapy or proton beam therapy. Each number reports one eye of one patient. The first 6 patients received ruthenium brachytherapy, while number 7 to 27 received proton beam therapy.

In the ruthenium group, mean visual acuity before endoresection in Snellen was 0.099 ± 0.197, which increased to 0.285 ± 0.302 after endoresection (p = 0.043). In the proton beam group, mean visual acuity before endoresection in Snellen was 0.030 ± 0.044, which decreased to 0.020 ± 0.036 after endoresection (p = 0.546). The changes in visual acuity for each individual patient is plotted in Figure 2.

Before radiation treatment, macular involvement of the tumor, retinal detachment or hemorrhage were present in 0 out of 6 patients in the ruthenium group, while this were 13 out of 21 patients in the proton group (p = 0.167). Before endoresection, macular involvement of the tumor, retinal detachment or hemorrhage were present in 3 out of 6 patients in the ruthenium group, while this were 21 out of 21 patients in the proton group (p < 0.001).

At last available visit, silicone oil was removed in 3 eyes (1 in the ruthenium group and 2 in the proton beam group), while oil was still present in 10 eyes (2 in the ruthenium group and 8 in the proton beam group).

## Discussion

This study comprises the outcomes of endoresection of uveal melanoma previously treated with ruthenium therapy or proton therapy.

A shorter duration between proton beam therapy and endoresection was observed, compared to ruthenium therapy and endoresection. This might be explained by the larger tumor size in the proton beam group, leading to more extensive radiation damage. Consequently, the likelihood of radiation-induced vitreoretinopathies is presumed to be lower in patients treated with ruthenium with a reduced risk of developing an indication for endoresection. However, tumor-specific characteristics such as a mushroom-shaped tumor, presence of exudates or hemorrhage, and location could also be of importance.

Embolisms have been described in choroidal melanoma during and after endoresection.^3,16,18^ It is hypothesized that air may be introduced into the venous system during fluid-air exchange via the vortex veins.^3^ Leakage of perfluorocarbon liquid into the circulatory system during endoresection has also been described, for which cauterization of the vortex veins in the quadrant corresponding to the tumor location has been suggested to avoid the risk of gas embolism.^19^ In our study, embolism was not observed in any of the patients. We hypothesize that the timing of surgery may be of relevance, with a greater regression of the tumor and decreased vascularization after a longer period of time after proton beam or ruthenium brachytherapy. A less vascularized tumor may lead to a lower chance of developing an embolism. Conversely, one may want to perform relatively prompt treatment in selected cases of retinal detachments in order to optimize visual outcomes, especially in cases without macular tumor involvement. However, this hypothesis is difficult to test due to the paucity of cases and retrospective nature of this study. Other limitations include the selection bias between the groups regarding tumor size (with smaller tumors in the ruthenium group). Therefore, comparisons between the groups are of limited value.

The results regarding visual outcomes are favorable in the ruthenium group, with a significant improvement in visual acuity after endoresection. For those treated with proton beam however, there was a decrease in visual acuity, albeit not significant. The visual outcome is presumed to be closely related to the underlying pathology, such as whether the tumor or radiation therapy involved the macula or optic nerve, and the intrinsic factors of the tumor.^11,20^ In such cases, improvement of visual acuity after endoresection is unlikely. In our study, we found that half of the patients treated with ruthenium and all of the patients treated with proton therapy, had tumor involvement of the macula, retinal detachment or hemorrhage in the macula before endoresection. The presence of silicone oil at last visit in 8 eyes in the proton beam group may also contribute to a decrease in visual acuity. In these cases however, retinal detachment was thought to be likely to occur if oil was removed. In addition, there are cases with a relatively short available follow-up. Visual acuity may still improve in the upcoming months.

In conclusion, adequate counselling of the patient is important before performing endoresection in uveal melanoma previously treated with ruthenium brachytherapy or proton beam therapy, as there is a wide variety in expected outcomes. Visual outcomes appear to be closely related to macular or optic disc involvement. We found a significant improvement in visual acuity after endoresection in the ruthenium group. In the proton beam group however, pain-free preservation of the eye may be the anticipated treatment outcome.

## Supplemental Material

**Figure:** Video 1.

## References

[1] Hussain RN Chiu A Pittam B , et al. Proton beam radiotherapy for choroidal and ciliary body melanoma in the UK-national audit of referral patterns of 1084 cases. Eye (Lond) 2023; 37: 1033–1036. 35840716 10.1038/s41433-022-02178-0 PMC10050435

[2] Damato B Groenewald C McGalliard J , et al. Endoresection of choroidal melanoma. Br J Ophthalmol 1998; 82: 213–218. 9602614 10.1136/bjo.82.3.213 PMC1722501

[3] Joussen AM Wong D . Egress of large quantities of heavy liquids from exposed choroid: a route for possible tumor dissemination via vortex veins in endoresection of choroidal melanoma. Graefe's Arch Clin Exp Ophthalmol 2015; 253: 177–178. 25572354 10.1007/s00417-014-2911-0

[4] Biewald E Lautner H Gök M , et al. Endoresection of large uveal melanomas: clinical results in a consecutive series of 200 cases. Br J Ophthalmol 2017; 101: 204–208. 27121095 10.1136/bjophthalmol-2015-307076

[5] Bechrakis NE Foerster MH . Neoadjuvant proton beam radiotherapy combined with subsequent endoresection of choroidal melanomas. Int Ophthalmol Clin 2006; 46: 95–107. 16365558 10.1097/01.iio.0000195856.31324.00

[6] Bechrakis NE Höcht S Martus P , et al. Endoresection following proton beam irradiation of large uveal melanomas. Ophthalmologe 2004; 101: 370–376. 15067418 10.1007/s00347-003-0911-2

[7] Schilling H Bornfeld N Talies S , et al. Endoresection of large uveal melanomas after pretreatment by single-dose stereotactic convergence irradiation with the leksell gamma knife–first experience on 46 cases. Klin Monbl Augenheilkd 2006; 223: 513–520. 16804822 10.1055/s-2006-926654

[8] O'Day RFJ Roelofs KA Negretti GS , et al. Long-term visual outcomes after ruthenium plaque brachytherapy for posterior choroidal melanoma. Eye (Lond) 2023; 37: 959–965. 35140328 10.1038/s41433-022-01944-4 PMC10050407

[9] Damato B Patel I Campbell IR , et al. Visual acuity after ruthenium(106) brachytherapy of choroidal melanomas. Int J Radiat Oncol Biol Phys 2005; 63: 392–400. 15990248 10.1016/j.ijrobp.2005.02.059

[10] Jung SK Park YH Shin DH , et al. Visual outcomes of proton beam therapy for choroidal melanoma at a single institute in the Republic of Korea. PLoS One 2020; 15: e0242966. 10.1371/journal.pone.0242966 PMC7710050 33264363

[11] Marinkovic M Horeweg N Fiocco M , et al. Ruthenium-106 brachytherapy for choroidal melanoma without transpupillary thermotherapy: similar efficacy with improved visual outcome. Eur J Cancer 2016; 68: 106–113. 27741435 10.1016/j.ejca.2016.09.009

[12] Patel AV Lane AM Morrison MA , et al. Visual outcomes after proton beam irradiation for choroidal melanomas involving the fovea. Ophthalmology 2016; 123: 369–377. 26545316 10.1016/j.ophtha.2015.09.031

[13] Melia BM Abramson DH Albert DM , et al. Collaborative ocular melanoma study (COMS) randomized trial of I-125 brachytherapy for medium choroidal melanoma. I. Visual acuity after 3 years COMS report no. 16. Ophthalmology 2001; 108: 348–366. 11158813 10.1016/s0161-6420(00)00526-1

[14] Marinkovic M Horeweg N Laman MS , et al. Ruthenium-106 brachytherapy for iris and iridociliary melanomas. Br J Ophthalmol 2018; 102: 1154–1159. 29122824 10.1136/bjophthalmol-2017-310688

[15] Ruschen H Romano MR Ferrara M , et al. Perfluorocarbon syndrome-a possible, overlooked source of fatal gas embolism following uveal-melanoma endoresection. Eye (Lond) 2022; 36: 2348–2349. 35352011 10.1038/s41433-022-02021-6 PMC9674629

[16] Rice JC Liebenberg L Scholtz RP , et al. Fatal air embolism during endoresection of choroidal melanoma. Retinal Cases and Brief Reports 2014; 8: 127–129. 25372327 10.1097/ICB.0000000000000021

[17] Morris RE Sapp MR Oltmanns MH , et al. Presumed air by vitrectomy embolisation (PAVE) a potentially fatal syndrome. Br J Ophthalmol 2014; 98: 765–768. 23793850 10.1136/bjophthalmol-2013-303367 PMC4033178

[18] Rojanaporn D Tipsuriyaporn B Chulalaksiriboon P , et al. Fatal air embolism after choroidal melanoma endoresection without air infusion: a case report. Ocul Oncol Pathol 2021; 7: 321–325. 34722487 10.1159/000518976 PMC8531826

[19] Fiorentzis M Bechrakis NE . Vortex vein cauterization and truncation to avoid perfluorocarbon syndrome during endoresection of uveal melanomas: a retrospective study. Eye (Lond) 2022; 37: 189–190. 35637346 10.1038/s41433-022-02119-x PMC9829721

[20] Marinkovic M Pors LJ van den Berg V , et al. Clinical outcomes after international referral of uveal melanoma patients for proton therapy. Cancers 2021; 13: 6241. 34944862 10.3390/cancers13246241 PMC8699723

